# Sleep architecture disturbance due to the use of benzodiazepines

**DOI:** 10.1192/j.eurpsy.2024.1614

**Published:** 2024-08-27

**Authors:** T. Jupe, K. Provi, I. Giannopoulos

**Affiliations:** Psychiatric Hospital of Attica, Athens, Greece

## Abstract

**Introduction:**

Insomnia, which is characterized by difficulty in initiating or maintaining a physiological sleep, is a relevant clinical issue, affecting not only the elderly population (from 20% to 40%), but also the general population since 30% of adults report problems in sleeping properly. In addition, 30-40% of adults report complaints about sleep quality lifetime, and10-15% report chronic insomniaBenzodiazepines (BZDs) are commonly prescribed to treat insomnia and sleep disorders. BZDs show a rapid sedative and anxiolytic effect, successfully used in the acute treatment of insomnia as well as anxiety, agitation, or anxiety associated with any type of psychiatric disorder. Their use is associated with potential side effects such as residual daytime sleepiness, ataxia, and dizziness. Long-term BZDs use may lead to drug abuse, tolerance, drug dependency, and abstinence. For instance, BZDs abrupt withdrawal can lead to severe symptoms such as insomnia and/or rebound anxiety, an increase in heart rate and blood pressure, nausea and/or vomiting, sweating, diarrhea, convulsions, and other neurological and psychiatric symptoms.

**Objectives:**

This e-poster aimed to summarize evidence regarding the effect of BZDs treatment on human Sleep Architecture.

**Methods:**

A bibliopgraphical review was performed using PubMed platform. All relevant articles were found using the keywords: benzodiazepines, sleep architecture, insomnia.

**Results:**

Prolonged use of benzodiazepines leads to an increase of time spent in stages 2 and a decrease of time in stages 1, 3, and 4. The increased NREM stage 2 is associated with a subjective improvement in sleep quality. The decrease in NREM sleep time in stages 3 and 4 is usually associated with lesser “rest” for the brain, which leads to a lack of concentration.

**Conclusions:**

BZDs use modified sleep architecture in the short and long term.

**Disclosure of Interest:**

None Declared

